# Sex-Dependent Changes in Right Ventricular Gene Expression in Response to Pressure Overload in a Rat Model of Pulmonary Trunk Banding

**DOI:** 10.3390/biomedicines8100430

**Published:** 2020-10-19

**Authors:** Hicham Labazi, Julie Birkmose Axelsen, Dianne Hillyard, Margaret Nilsen, Asger Andersen, Margaret R. MacLean

**Affiliations:** 1 Strathclyde Institute of Pharmacy and Biomedical Sciences, University of Strathclyde, Glasgow G4 0RE, UK; hicham.labazi@strath.ac.uk (H.L.); margaret.nilsen@strath.ac.uk (M.N.); 2 Institute of Cardiovascular and Medical Sciences and College of Medical, Veterinary and Life Sciences, University of Glasgow, Glasgow G12 8QQ, UK; Dianne.Hillyard@uws.ac.uk; 3 Department of Cardiology, Aarhus University Hospital, 8200 Aarhus, Denmark; julieaxelsen@clin.au.dk (J.B.A.); asger.andersen@clin.au.dk (A.A.)

**Keywords:** pulmonary hypertension, pulmonary trunk banding, right ventricular hypertrophy, compensated right ventricular failure, fibrosis, sexual differences

## Abstract

Right ventricular hypertrophy (RVH) and subsequent failure are consequences of pulmonary arterial hypertension (PAH). While females are four times more likely to develop PAH, male patients have poorer survival even with treatment, suggesting a sex-dependent dimorphism in right ventricular (RV) hypertrophy/compensation. This may result from differential gene expression in the RV in male vs. female. To date, the sex dependent effect of pressure overload on RV function and changes in gene expression is still unclear. We hypothesize that pressure overload promotes gene expression changes in the RV that may contribute to a poorer outcome in males vs. females. To test this hypothesis, male and female Wistar rats underwent either a sham procedure (sham controls) or moderate pulmonary trunk banding (PTB) (a model of pressure overload induced compensated RV hypertrophy) surgery. Seven weeks post-surgery, RV function was assessed in vivo, and tissue samples were collected for gene expression using qPCR. Compared to sham controls, PTB induced significant increases in the right ventricular systolic pressure, the filling pressure and contractility, which were similar between male and female rats. PTB resulted in an increase in RVH indexes (RV weight, RV weight/tibia length and Fulton index) in both male and female groups. However, RVH indexes were significantly higher in male-PTB when compared to female-PTB rats. Whilst end of procedure body weight was greater in male rats, end of procedure pulmonary artery (PA) diameters were the same in both males and females. RV gene expression analysis revealed that the following genes were increased in PTB-male rats compared with the sham-operated controls: natriuretic peptide A (ANP) and B (BNP), as well as the markers of fibrosis; collagen type I and III. In females, only BNP was significantly increased in the RV when compared to the sham-operated female rats. Furthermore, ANP, BNP and collagen III were significantly higher in the RV from PTB-males when compared to RV from PTB-female rats. Our data suggest that pressure overload-mediated changes in gene expression in the RV from male rats may worsen RVH and increase the susceptibility of males to a poorer outcome when compared to females.

## 1. Introduction

Pulmonary arterial hypertension (PAH), defined as a mean pulmonary arterial pressure of >20 mmHg at rest, is characterized by increased pulmonary vascular resistance, progressive remodeling and obstruction of distal pulmonary resistance arteries causing increased right ventricle (RV) afterload [1]. To maintain sufficient cardiac output (CO) to meet the body’s metabolic demand, the thin-walled RV undergoes hypertrophy as an adaptive mechanism when faced with the pressure overload. Over time, the increasing RV afterload results in a declined RV function and a development of right heart failure (RHF), culminating in death [2]. In PAH, RV function is the most important predictor of the disease morbidity and mortality [3]. The incidence of PAH is significantly higher in females than males, making female sex a clinically significant risk factor for the development of PAH [4,5,6,7]. Conversely, male PAH patients have significantly poorer outcomes than females, making male sex an independent predictor of worse survival in PAH [8,9,10]. This sexual dichotomy in the development, pathogenesis and progression of the disease suggests a potential role of sex and sex hormones in the disease. In fact, Kawut et al. have shown that healthy women have better RV function, in particular higher RV ejection fraction compared to healthy men [11]. In addition, they showed that women have lower RV mass and volumes than men [8]. Cardiac adaptation to PAH therapy associated with long-term improvement in RV ejection fraction was observed in women but not in men [8,12]. Pulmonary artery banding was shown to increase RV fibrosis in male mice, while testosterone deprivation by castration appears to reduce RVH and improve survival in these male mice, without significantly affecting RV function and hemodynamics [13]. These studies suggest that sex/sex hormones are key factors in RV structure and function.

To date, there are no therapies which directly target the RV remodeling in pulmonary hypertension (PH). Instead, most therapies aim to reduce afterload, optimize preload and increase RV contractility. In fact, in experimental models of PH, the RV failure is secondary to disease in the pulmonary vasculature. As a consequence, the improvement of RV function in response to current therapy may be secondary to afterload reducing pulmonary vascular effects rather than its direct effects on the RV. Contrary to other models of PAH (such as Sugen and monocrotaline-induced PAH), the pulmonary trunk banding model can allow for the investigation of direct cardiac effects of an intervention on the RV, independently of the pulmonary vasculature. To date, no study has investigated sex differences in early RV gene expression in this model; our study aims to compare and investigate differential RV response to pressure overload in male vs. female rats [14].

## 2. Materials and Methods

### 2.1. Animals

Four week old male and female Wistar rats (109 g ± 9 g, Janiver Labs, Hannover) were given free access to water and standard rat chow (Altromin #1324; Altromin, Lage, Germany). Two animals per cage were housed in a room with a 12-h light-dark cycle and a temperature of 23 °C given a small tunnel and a piece of wood to chew. The rats were treated according to Danish national guidelines, and all experiments were approved by the Institutional Ethics Review Board and conducted in accordance with the Danish Law for animal research (authorization number 2016-15-0201-01040, Danish Ministry of Justice, approval date 10 October 2016).

### 2.2. Study Design

Compensated RV hypertrophy was induced by pulmonary trunk banding (PTB). A group of male rats and a group of female rats were randomized to either sham operation or PTB, resulting in four groups: a male sham group (*n* = 6), a male PTB group (*n* = 10), a female sham group (*n* = 6) and a female PTB group (*n* = 10). In total, 32 rats were included in the study. Seven weeks after the surgery, RV function was evaluated by echocardiography and invasive pressure-volume measurements. Afterwards, the rats were euthanized, the hearts excised and molecular analyses were performed to asses RV remodelling.

### 2.3. Pulmonary Trunk Banding

The rats were anesthetized with sevofluranein 2:1 O_2_/air mix (7% induction and 3.5% maintenance), intubated and ventilated (Abbot Scandinavia, Solona, Sweden; RF 76 min^−1^ and tidal volume 1.5 mL). The rats were injected with buprenorphine s.c. (0.1 mg/kg, Temgesic, Indivior UK Limited, Hull, UK), shaved on the thorax, and a lateral thoracotomy was performed. The pulmonary trunk was carefully isolated, and the banding was made with a modified horizon applier that compressed the titanium clip to a pre-set inner diameter of 0.7 mm. The rats were injected with 2 mL s.c. isotonic saline solution to compensate for fluid loss, and the thorax was closed in three layers. As additional analgesics, the rats received s.c. carprofen (5 mg/kg, Norodyl Vet, ScanVet Animal Health, Fredensborg, Denmark). To relieve postoperative pain, the rats were treated with buprenorphine in the drinking water (7.4 µg/mL) for 3 days. Except for the application of the clip around the pulmonary trunk, sham operated animals underwent the same procedure [15].

### 2.4. Evaluation of Hemodynamic

All rats underwent transthoracic echocardiography under general anaesthesia (spontaneously breathing, sevoflurane (in 2:1 O_2_/air mixwith 7% induction and 3.5% maintenance) [16]. The thoraces of the rats were shaved and remaining hair removed with Veet^®^. A MS250 line array transducer on a Vevo 2100 imaging System (version 1.5.0) (VisualSonics Inc., Toronto, ON, Canada) was used scanning at a frequency of 14–21 MHz. Heart rate (HR) was obtained through extremity electrodes. Tricuspid annular plane systolic excursion (TAPSE) was measured in an apical 4-chamber view. In the parasternal long axis view, we measured the velocity time integral (VTI) at three systematically chosen points in the pulmonary trunk using the pulsed wave and colour Doppler. The systolic diameter of the pulmonary trunk was measured, and stroke volume (SV) computed as: $\mathrm{SV}={\left(\frac{\mathrm{PT}\;\mathrm{diameter}}{2}\right)}^{2}\cdot \pi \cdot \mathrm{VTI}$. All images were analysed off-line (Vevo^®^ 2100, Fujifilm VisualSonics Inc., Amsterdam, The Netherlands) with the observer blinded to the source of the sample. To average beat-to-beat variation, all parameters were measured in three consecutive heart cycles.

For the invasive pressure-volume measurements, the rats were anesthetized with sevofluranein 2:1 O_2_/air mix (7% induction, 3.5% maintenance), intubated and ventilated (Abbot Scandinavia, Solona, Sweden; RF 76 min^−1^ and tidal volume 4.5 mL). To prevent blood clotting during the procedure, the rats were injected intramuscularly with 50 units of heparin (Heparin, Leo Pharma A/S, Ballerup, Denmark). We measured systemic blood pressures with a pressure catheter (SPR-320, Millar Instruments, Houston, TX, USA) installed in the left carotid artery after stabilization. A conductance catheter (SPR-869, Millar Instruments, Houston, TX, USA) installed in the RV through the apex obtained RV pressure-volume loops. Signals were sampled by MPVS Ultra (Millar Instruments) and processed in Powerlab 16/35 (AD Instruments, Oxford, UK). Steady state RV pressures were recorded after stabilization. Consecutive pressure-volume loops were produced by simultaneous recordings of RV pressures and volumes with decreasing preloads caused by slowly occluding the inferior vena cava. Load-independent measures of diastolic function and RV contractility (e.g., ventriculo-arterial coupling and end-systolic elastance (Ees) were calculated from the pressure-volume loops using LabChart Software (AD Instruments, UK). The conductance signal was calibrated using SV derived from echocardiography measurements.

### 2.5. Evaluation of Anatomic Measures

The heart was quickly excised and the RV separated from the left ventricle (LV)+ septum and weighed. RV tissue was snap frozen for molecular analyses. Remaining organs were weighed. The left lung was inflated with neutralised buffered formalin and a small piece of the right lung was snap frozen for molecular analyses.

### 2.6. Measurement of Right Ventricular Hypertrophy

Right ventricular hypertrophy (RVH) or Fulton index was assessed as the weight of the RV free wall/the weight of the LV + septum (Fulton Index = RVH = RV/(LV + septum). Another way of assessing RVH is the ratio of RV weight to the tibia length (RV/tibia).

### 2.7. Gene Expression

Lung and RV tissue were harvested and were stored at −80 °C until RNA isolation was performed. Lung and RV tissues were lysed using a Tissue Lyser (Qiagen). Total RNA was extracted using the QIAGEN miRNeasy mini-kit (Qiagen, Manchester, UK) following the manufacturer’s instructions. Treatment with DNAse 1 (Qiagen) eliminated genomic DNA contamination prior to quantification using a NanoDrop ND-1000 Spectrophotometer (Nano-Drop Technologies, Wilmington, DE, USA). RNA was then reverse transcribed to cDNA using the TaqMan Reverse Transcription kits (Life Technologies, Paisley, UK). The mRNA expression was assessed using TaqMan Gene Expression probes (Life Technologies, Paisley, UK) by quantitative real-time polymerase chain reaction (qRT-PCR) and normalized to a housekeeper β-2-microglobulin (B2M). TaqMan assay IDs are presented in Table 1.

### 2.8. Pulmonary Artery Remodeling

This was carried out to check that the pulmonary vasculature was unaffected by the banding procedure. Five micrometres of lung sagittal sections were stained with elastin/Picro Sirius red for identification of vascular remodeling. Pulmonary arteries (80 to 100 arteries per lung) from the four experimental groups (*n* = six each group) were microscopically assessed for degree of muscularisation in a blinded fashion. Remodeled arteries were confirmed by the presence of double-elastic laminae, and percentage remodeling (percent of remodeled vessels) was defined for each animal by the number of remodeled vessels divided by the total number of vessels observed in the lung (>80 vessels). Images were captured using a Zeiss Axio Imager M1.

### 2.9. Statistical Analysis

Pressure, hemodynamic and gene expression data were analyzed using one-way ANOVA with Bonferroni post hoc analysis. All graphs and statistical analyses were produced and performed using GraphPad Prism. Data are expressed as means ± S.E.M. (*n*), where ‘n’ is the number of rats. Values of *p* < 0.05 were considered statistically significant.

## 3. Results

### 3.1. Effect of Sex and PTB on Physiological Parameters

**Figure 1:**
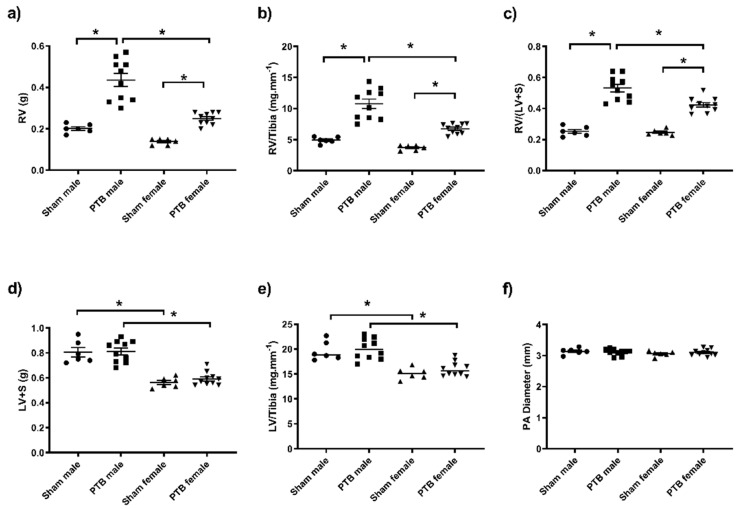
The cardiovascular anatomical changes in response to PTB surgery in male and female rats. (**a**) RV weight, (**b**) RV weight/tibia length, (**c**) RV weight/(LV + S) weight, (**d**) LV + S weight, (**e**) LV weight/ tibia length and (**f**) PA diameter. RV: right ventricle, LV: left ventricle, LV + S: left ventricle + septum, PA: pulmonary artery. Sham male (*n* = 6), BTP male (*n* = 10), sham female (*n* = 6) and PTB female (*n* = 10). Data expressed as mean ± SEM with * *p* < 0.05.

The initial body weight of both male and female was not different between all groups. At the end of the study, the body weight was significantly higher in males compared to female rats; however, PTB surgery did not affect the body weight in the same sex (Table 2). Similar to body weight, females (both sham and PTB) had significantly lower tibia length, as well as liver, lung, spleen and kidney weights when compared to males (Table 2). RV weight, RV/tibia and Fulton index (FI) were significantly increased as a result of PTB surgery in both males and females (Figure 1a–c). While the RV weight, RV/tibia and FI were not different between the sham groups, these parameters were significantly higher in PTB males compared to PTB females (Figure 1a–c). The LV weight and LV/tibia ratio were not affected by PTB surgery; however, they were both significantly higher in males compared to females (Figure 1d,e). Despite the difference in body weight between male and female groups, the diameter of the PA was not significantly different between the four experimental groups (Figure 1f).

### 3.2. Effect of Sex and PTB on Cardiac Function

**Figure 2:**
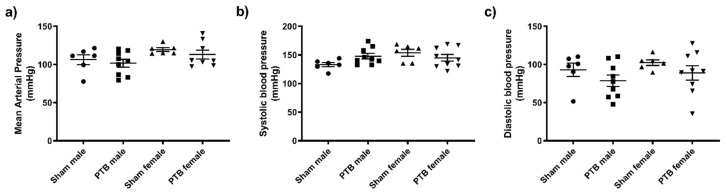
Effect of PTB surgery on (**a**) mean arterial pressure, (**b**) systolic blood pressure and (**c**) diastolic blood pressure. Sham male (*n* = 6), BTP male (*n* = 10), sham female (*n* = 6) and PTB female (*n* = 10). Data expressed as mean ± SEM.

**Figure 3:**
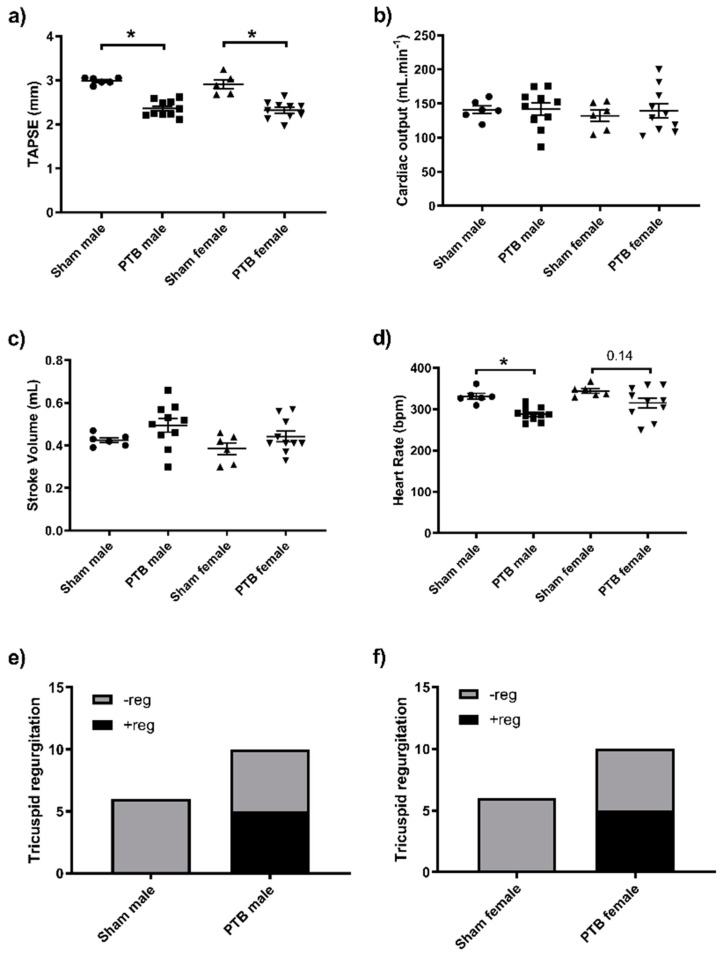
Echocardiography: effect of PTB surgery on (**a**) Tricuspid annular plane systolic excursion (**b**) cardiac output (**c**) stroke volume (**d**) heart rate and (**e**,**f**) Tricuspid regurgitation. Sham male (*n* = 6), BTP male (*n* = 10), sham female (*n* = 6) and PTB female (*n* = 10). Data expressed as mean ± SEM with * *p* < 0.05.

**Figure 4:**
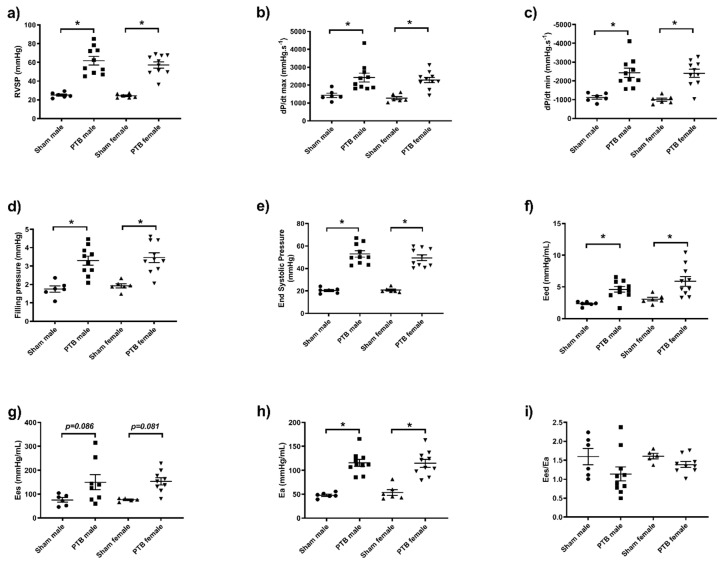
Effect of PTB surgery on right ventricle (RV) function in male and female rats. (**a**) RVSP: right ventricular systolic pressure, (**b**,**c**) RV contractility: negative and positive *dp*/*dt*, (**d**) filling pressure, (**e**) end systolic pressure, (**f**) Eed: end-diastolic elastance, (**g**) Ees: end-systolic elastance, (**h**) Ea: arterial elastance, (**i**) Ees/Ea: ventriculo-arterial coupling. Sham male (*n* = 6), BTP male (*n* = 10), sham female (*n* = 6) and PTB female (*n* = 10). Data expressed as mean ± SEM with * *p* < 0.05.

Systolic, diastolic and mean arterial pressure were not affected by either sex or PTB surgery (Figure 2). Tricuspid annular plane systolic excursion (TAPSE) was not different between sham males and sham females; however, it was equally reduced in both PTB-males and PTB-females (Figure 3a). Both CO and SV were not affected by the rat’s sex or PTB surgery (Figure 3b,c). PTB caused a significant decrease in HR in male rats, but it was not significant between sham and PTB female rats (*p* = 0.14) (Figure 3d). In both male and female rats with PTB, Tricuspid regurgitation (TR) occurred in half of the animals in each group (Figure 3e,f). PTB surgery caused a significant increase in right ventricular systolic pressure (RVSP), filling pressure and contractility (*dp*/*dt* max and *dp*/*dt* min) (Figure 4a–d), which were not affected by sex. End systolic pressure (ESP) was not affected by animal’s sex, yet, it was significantly increased as result of PTB in both male and female rats (Figure 4e). The RV’s end systolic elastance (Ees) tended to increase following PTB, but it was not significant in both male and female rats (Figure 4g). Arterial elastance (Ea) (Figure 4h) and end-diastolic elastance (Eed) (Figure 4f) were both increased by PTB surgery, whereas sex did not affect both parameters in sham or PTB groups. The ventriculo-arterial coupling (Ees/Ea) was not effected by either sex or PTB surgery (Figure 4i).

### 3.3. Effect of PTB on RV Gene Expression

**Figure 5:**
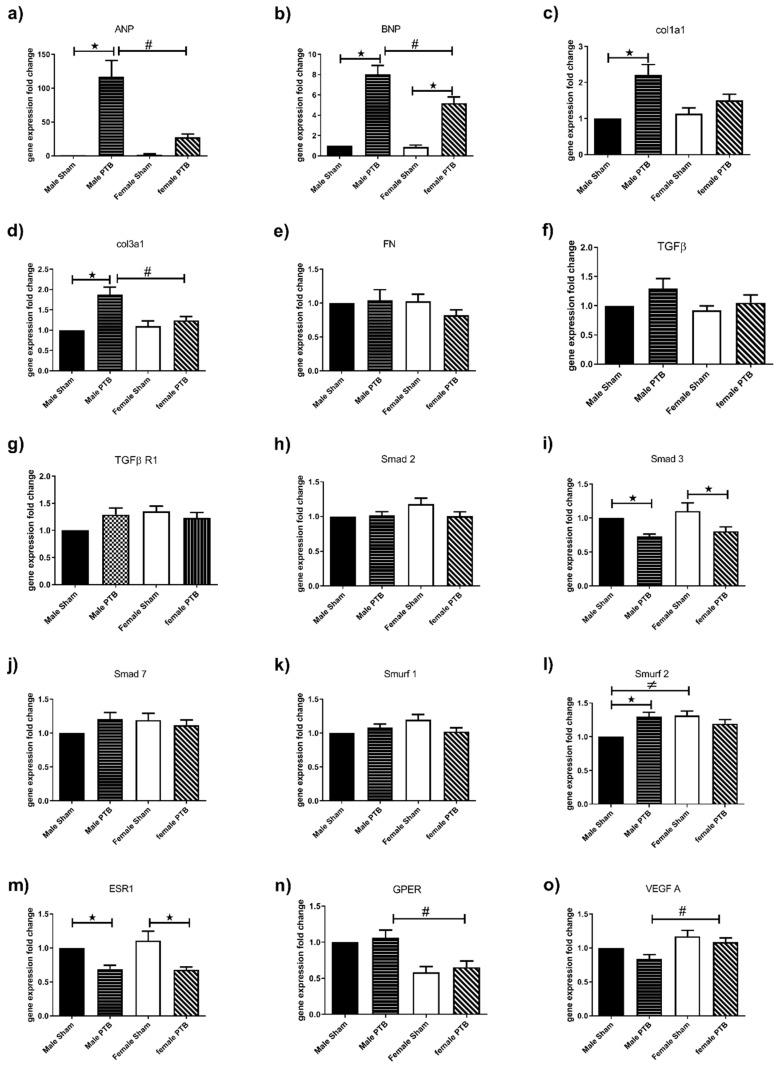
The effect of pulmonary trunk banding RV’s gene expression. For gene expression by quantitative real time polymerase chain reaction (PCR), total RNA was extracted from rat’s RV samples from male sham (*n* = 6), female sham (*n* = 6), male BTP (*n* = 10) and female PTB (*n* = 10) groups. (**a**) ANP (Natriuretic peptide type A), (**b**) BNP (Natriuretic peptide type B), (**c**) col1a1 (Collagen 1), (**d**) col3a1 (Collagen 3), (**e**) FN (Fibronectin), (**f**) TGF-β (Transforming growth factor-β,) (**g**) TGF-βR1 (Transforming growth factor-β receptor 1), (**h**) Smad 2 (SMAD family member 2), (**i**) Smad 3 (SMAD family member 3), (**j**) Smad 7 (SMAD family member 7), (**k**) Smurf 1 (SMAD specific E3 ubiquitin protein ligase 1), (**l**) Smurf 2 (SMAD specific E3 ubiquitin protein ligase 2), (**m**) ESR1 (Estrogen receptor 1), (**n**) GPER (G protein-coupled estrogen receptor) and (**o**) VEGF A (Vascular endothelial growth factor A). β-2microglobulin was used as the internal reference gene, and data were expressed as fold change of the male sham group. Data expressed as mean ± SEM. ^★^ *p* < 0.05 Sham vs. BTP in the same sex, ^≠^ *p* < 0.05 Sham-male vs. Sham-female and # *p* < 0.05 BTP-male vs. PTB-female.

*Hypertrophy.* mRNA analysis of genes associated with RV hypertrophy showed that ANP was only significantly increased in PTB-males but not females. Furthermore, ANP was significantly higher in the RV from PTB-males compared to that of the PTB-females (Figure 5a). PTB caused a significant increase in BNP expression in both male and female rats. Similar to ANP, BNP was significantly higher in the RV from PTB-males compared to that of the PTB-females (Figure 5b).

*Fibrosis.* mRNA analysis of genes associated with fibrosis indicated that PTB surgery induced significant increases in collagen 1 (Col1) and 3 (Col3) gene expression in males, but not in females. Furthermore Col3 gene expression was significantly higher in PTB males compared to PTB females (Figure 5c,d). Neither PTB nor sex had an effect on the expression of the fibronectin (FN) gene (Figure 5e).

*TGFβ signaling.* While TGFβ and its receptor TGFβR1 were not changed, we observed only decreased expression of Smad3 gene in both male and female PTB rats when compared to the corresponding sham (Figure 5i), while the expression of Smurf2 was only increased in PTB-males compared to sham-males (Figure 5l).

*Estrogen receptors.* mRNA analysis of genes associated with estrogen receptors showed a decreased estrogen receptor α (ESR1) genes expression in both males and females following PTB surgery (Figure 5m). Estrogen receptor β (ESR2) was not detectable, while G protein-coupled estrogen receptor (GPER) gene expression was significantly decreased in female PTB-female rats when compared to PTB-male rats (Figure 5n).

PTB-female’s RV expressed higher VEGF gene expression compared to that of the PTB male rats (Figure 5o).

### 3.4. Effect of PTB on Lung Gene Expression

**Figure 6:**
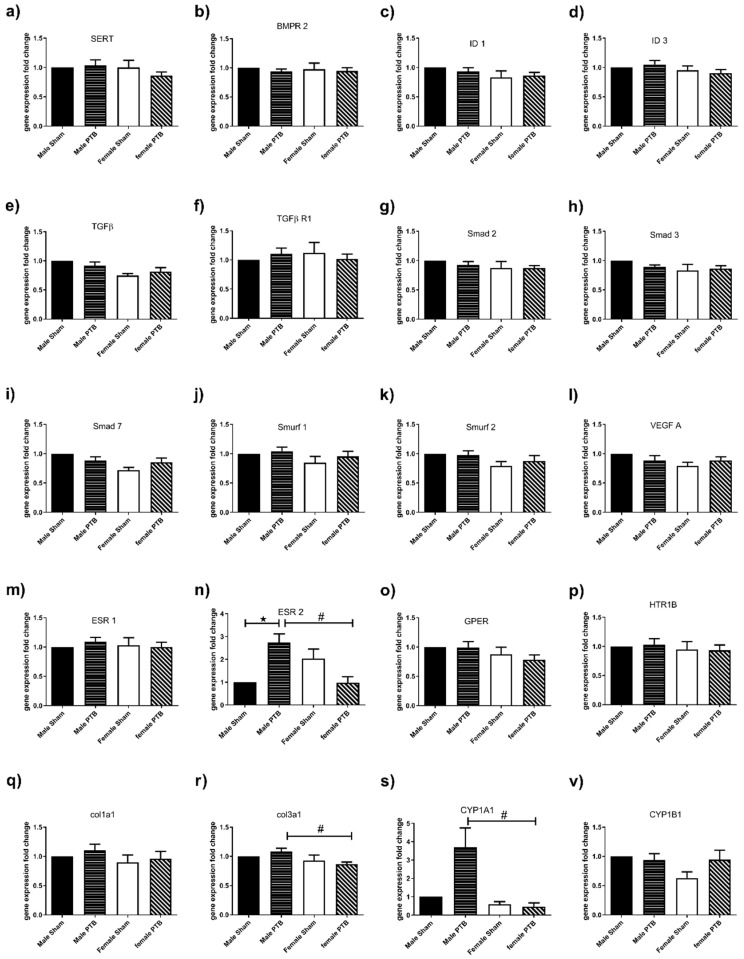
The effect of pulmonary trunk banding (PTB) on the lung’s gene expression. For gene expression by quantitative real time PCR, total RNA was extracted from rat’s lung samples from male sham (*n* = 6), female sham (*n* = 6), male BTP (*n* = 10) and female PTB (*n* = 10) groups. (**a**) SERT (Serotonin transporter), (**b**) BMPR 2 (bone morphogenetic protein receptor, type II), (**c**) ID 1 (inhibitor of DNA binding 1), (**d**) ID 3 (inhibitor of DNA binding, 3) (**e**) TGF-β(Transforming growth factor-β), (**f**) TGF-βR1 (Transforming growth factor-β receptor 1), (**g**) Smad 2 (SMAD family member 2), (**h**) Smad 3 (SMAD family member 3), (**i**) Smad 7 (SMAD family member 7), (**j**) Smurf 1 (SMAD specific E3 ubiquitin protein ligase 1), (**k**) Smurf 2 (SMAD specific E3 ubiquitin protein ligase 2), (**l**) VEGF A (Vascular endothelial growth factor A), (**m**) ESR1 (Estrogen receptor 1), (**n**) ESR2 (Estrogen receptor 2), (**o**) GPER (G protein-coupled estrogen receptor), (**p**) HTR1B (5-hydroxytryptamine (serotonin) receptor 1B), (**q**) col1a1 (Collagen 1), (**r**) col3a1 (Collagen 3), (**s**) CYP1A1 (cytochrome P450, family 1, subfamily a, polypeptide 1) and (**v**) CYP1B1 (Cytochrome P450, family 1, subfamily b, polypeptide 1). β-2microglobulin was used as internal reference gene, and data were expressed as fold change compared of the male sham group. Data expressed as mean ± SEM. ^★^ *p* < 0.05 Sham vs. BTP in the same sex and ^#^ *p* < 0.05 BTP-male vs. PTB-female.

PTB is a model of pressure overload, which is independent of changes in pulmonary vasculature and lungs. As expected, most genes were not affected by PTB surgeries in males and females. However, ESR2 expression was significantly increased in lungs from PTB-males compared to sham males (Figure 6n). Furthermore, lungs from PTB-females had significantly lower gene expression of ESR2, Col3a1 and CYP1A1 genes compared to PTB-males (Figure 6n,r,s).

### 3.5. Effect of PTB on Lung’s Microvascular Remodeling

As expected, the PTB did not affect microvascular remodeling in both males and females. No remodeling was observed in the lungs from the four experimental groups.

## 4. Discussion

In this study, we compared the RV response to increased cardiac afterload following moderate PTB between male and female rats. The degree of banding (moderate) caused compensatory RV remodeling with preserved CO and SV in both males and females, although the degree of hypertrophy was significantly higher in male vs female rats. TAPSE was equally reduced in males and females. In both sexes, PTB banding also resulted in similar increases in RVSP, RV filling pressure, contractility (*dp*/*dt*), arterial elastance and end-diastolic elastance, while the ventriculo-arterial coupling was not changed as a result of PTB. This demonstrates that, despite no sex differences in the degree of PH and RV dysfunction, the degree of ventricular hypertrophy was much greater in the males’ RV. The RVH was associated with a significant increase in RV hypertrophy markers gene expression (ANP and BNP), as well as fibrosis genes (Col1 and 3) in male when compared to female rats.

The PTB model is a model of increased RV afterload, which is observed in PAH and some congenital heart diseases [17]. In adaptation to the increased afterload in disease states such as PH, RV hypertrophies to maintain a physiological CO and SV. If left untreated, there is transition to decompensated RV hypertrophy, ensuing RHF associated with decreased CO and SV, eventually culminating in death. In PAH, despite current vasodilator therapies, RHF is an important cause of mortality. This may result from the fact that these treatments target pulmonary vascular dysfunction, which may result in an improvement of RV function, which may be secondary to decreased RV afterload. So, it is imperative to develop cardioprotective treatments which aim primarily to improve RV function, especially because patients often present in clinic at a later stage of the disease, when the RV dysfunction has been established [14]. Benefits of using a PTB model includes investigation of RVH and RHF independent from changes in pulmonary microvascular tone or remodeling, as well as permitting development of therapeutics that aim to improve RV function independent of changes in the afterload.

In the present study, RVSP was increased in both males and females to a similar extent, so were the filling pressure and contractility. This increase in RVSP was associated with an increase in RV hypertrophy with preserved CO and SV, suggesting that an adaptive remodelling of the RV, which corroborate previous studies with rats subjected to mild PTB [18,19]. TAPSE was reduced to similar extent in both male and female rats following PTB procedure, as shown by others [18,19], suggesting an early RV dysfunction during the compensated RHF stage. In our study, both Fulton index (RV/LV + S) and RV weight/tibia length, which are both indices of RVH, were significantly higher in male rats when compared to female rats. This may indicate an advanced RHF in males compared to females, and corroborate a clinical study showing that male PAH patients have worse RV function despite similar afterload [20]. Of note, this difference in RVH was not due to the degree of restriction as both male and female rats were subjected to the same degree of banding (0.7 mm clip), nor the size of the PA since we did not observe any difference in the artery size at the end of the study. TR, which is an independent risk factor of transplantation and death in PAH patients [21], was present in both PTB groups (50% occurrence). Hemodynamic measurements showed an increase in end diastolic elastance (Eed) suggesting an onset of diastolic dysfunction [22]; however, this increase in Eed was not different between male and female PTB rats. PTB resulted in an increased arterial elastance (Ea) in both male and female rats, which was not significantly affected by sex. With the increase of afterload, the end diastolic elastance, an index of RV systolic function tended to increase both in male and female PTB rats, but it was not significant (*p* = 0.09 and *p* = 0.08 respectively). Although the Ees increase was not significant, it was enough to maintain the ventriculo-arterial coupling (Ees/Ea), confirming that the RVs in our study from moderate PTB (both male and female rats) were able to compensate for the increase in afterload. These hemodynamic changes due to PTB were not different between male and female-PTB rats, suggesting that despite the same hemodynamic measurements, at least at the compensated stage of the RHF, males may present advanced RV hypertrophy than females.

Gene expression analysis in the RV showed an increased ANP in RV from PTB-male rats, while PTB-female rats showed a trend to increase but it was not significant. Furthermore, RV’s ANP levels following PTB procedure showed a significant increase in PTB-male compared to PTB-female. ANP is a marker of ventricular hypertrophy that was shown to be increased in hypertrophied heart in response to increased pressure overload [23,24]. Our data corroborate a previous study showing that an increase of ANP gene expression in RV from male rat model of pressure overload, which was reversed by castration accompanied with reduced RVH and improved survival in these male mice [13]. Taken together, this suggests that ANP expression may be regulated by testosterone, contributing to increased RVH in males. In fact, early in vitro studies have demonstrated that testosterone induces increased ANP synthesis in neonatal male atrial and ventricular myocytes [25,26]. In response to wall stress resulting from volume or pressure overload, ventricular myocytes synthesize and secrete BNP. The BNP expression, which is associated with the degree of RV dysfunction [16,23], was increased in RVs from both PTB-male and PTB-female rats; however, BNP expression was significantly higher in PTB-males when compared to PTB-females. Our data suggest that PTB-females may exhibit a slower progression of hypertrophy and RV dysfunction. Interestingly, similar sexual dimorphism associated with better RV adaptation and less hypertrophy was also shown in other models of RV failure such as the monocrotaline and Sugen-induced pH [14].

As sex and sex hormones, mainly estrogen, were associated with better RV function, we examined estrogen receptor expression in the RV. While ESR1 was decreased to a similar extent in male and female-PTB animals, GPER was not affected by PTB surgery. However, GPER expression was significantly decreased in female-PTB compared to male-PTB. Our results are in agreement with a study showing that ESR1 and GPER are expressed at similar level in males’ and females’ hearts [27]. In our study, ESR2 was undetectable in the RV, which supports previous studies showing below detectable ESR2 gene in rodent’s cardiac tissue [27,28]. The decreased ESR1 expression in both male and female rats undergoing PTB procedure may contribute to RV dysfunction, as ESR1was shown to have a cardioprotective function [29]. RV fibrosis and changes in extracellular matrix genes expression, especially collagen was shown to play an important role in the disease progression [30]. Gene expression in RV from PTB rats shows that only PTB-males had a significant increase in collagen I and III genes, while no change in fibronectin gene expression was observed in both male and female-PTB rats. Furthermore, collagen III was significantly higher in PTB-males when compared to PTB-females. Our data suggest that RVs from PTB-male have increased fibrosis compared to female. Our data are in agreement with a previous study showing increased collagen in PTB-male rats [23,24]. Another important player in mediating cardiac hypertrophy and fibrosis is TGF-β signaling, and inhibition of TGF-βR1was shown to prevent cardiac fibrosis and hypertrophy [31,32,33,34]. TGF-β binds to its receptor leading to activation of Smad 2/3, which forms a complex with Smad4. The complex then translocates into the nucleus inducing transcription of genes associated with fibrosis and hypertrophy. In our study, while TGF-β and TGF-βR1 were not affected by sex or PTB banding, its downstream signaling elements such as Smad3 and Smurf2 were changed.

Smurf2 is a negative regulator of TGF-β signaling that targets Smad3 through multiple mono-ubiquitination, preventing its association with Smad4 and their translocation to the nucleus [35,36]. Smad3 was decreased in both male and female-PTB rats, while Smurf2 was only increased in male-PTB rats. Furthermore, the baseline expression of Smurf2 was significantly higher in RV from sham-female vs. sham-male rats. This decrease in Smad3 signaling may be a compensatory mechanism to limit the increase in fibrosis, at least at an early stage of RHF. VEGF expression was higher in PTB-females when compared to PTB-males. VEGF is an important player in controlling RV perfusion by decreased capillary rarefaction, thus slowing the transition from a compensated RVH to decompensated RVH [37,38]. Together, our RV gene expression data suggest that female sex might be cardioprotective by slowing the progression of RVH and fibrosis, and ultimately the transition into a decompensated heart. Our data may shed a light on possible mechanisms that predispose males to have the worst outcome in PAH despite a lower incidence rate compared to females. In the lung, we looked at the expression of genes associated with development of PAH in the lung. We did not observe any changes of genes in lungs from PTB-females. In PTB-males the only change was an increased ESR2 expression. Furthermore, lungs from PTB-males had a significant increase in ESR2, Col3 and CYP1A1 expression when compared to female rats. Increased collagen deposition and CYP1A1 activation is known to promote PAH [39,40], whereas ESR2 was shown to both induce and prevent PAH [14,41]. This discrepancy in the ESR2 role may be animal- (rats vs. mice) or disease-specific (inducible vs. genetic). Future study looking at the possible effect of PTB surgery on the expression and function of genes associated with PAH may be necessary to understand the effect of pressure overload on the lung/pulmonary microvessel function.

A couple of limitations of the study should be noted. First, the rats at the beginning of the study were relatively young (4 weeks), which introduces a possible effect of age. We know that our studies exhibited some sex differences, and so the effect of age and sex hormones over the course of the study may have contributed to the pathogenesis of pulmonary hypertension and RVH. Future studies investigating at the effect of PTB on the RV function and hypertrophy at later ages, especially considering the age of the human patients in the clinic, would add value to our understanding of the sex difference in the pathogenesis and the progression of the RVH and the RHF. Second, in the present study, we focused on gene expression changes in the lung and RV. In future studies, identification of changes in protein expression and post-translational modifications of the genes would increase our understanding of the pathophysiological effect of the pressure overload at the molecular level.

## 5. Conclusions

Our study is the first study to investigate the effect of sex on the RV structure and function combined with gene expression in a pressure overload model of compensated RHF. Compared to females and despite presenting similar hemodynamic measurements, males had higher RVH and increased expression of cardiac hypertrophy and fibrosis genes, which may explain the poorer outcome observed in males in pulmonary hypertension.

## Figures and Tables

**Table 1:** List of the TaqMan^®^ gene expression assay IDs used for the gene expression experiments.

Gene Name	Assay ID
B2m beta-2 microglobulin	Rn00560865_m1
Col1a1 collagen, type I, alpha 1	Rn01463848_m1
Cyp1b1 cytochrome P450, family 1, subfamily b, polypeptide 1	Rn04219389_g1
ID1 inhibitor of DNA binding 1	Rn00562985_s1
ID3 inhibitor of DNA binding 3	RN00564923_m1
NPPA natriuretic peptide type A	RN00664637_g1
NPPB natriuretic peptide type B	Rn00580641_m1
VEGFA	RN01511602_m1
Smurf 1 SMAD specific E3 ubiquitin protein ligase 1	Rn01412801_m1
Smurf 2 SMAD specific E3 ubiquitin protein ligase 2	Rn01452783_m1
Smad 2 SMAD family member 2	Rn00569900_m1
Smad 3 SMAD family member 3	Rn00565331_m1
Smad 7 SMAD family member 7	Rn01523958_m1
GPER G protein-coupled estrogen receptor 1	Rn01643280_s1
ESR 1 estrogen receptor 1 (alpha)	Rn01640372_m1
ESR 2 estrogen receptor 1 (beta)	Rn00562610_m1
Tgfbr1 transforming growth factor, beta receptor I	Rn00688966_m1
Htr1b 5-hydroxytryptamine (serotonin) receptor 1B	Rn01637747_s1
SERT Serotonin Transporter	Rn00564737_m1
Bmpr2 bone morphogenetic protein receptor, type II (serine/threonine kinase)	Rn01437214_m1
Cyp1a1 cytochrome P450, family 1, subfamily a, polypeptide 1	Rn00487218_m1
Fn1 fibronectin 1	Rn00569575_m1
Col3a1 collagen, type III, alpha 1	Rn01437681_m1
Tgfb1 transforming growth factor, beta 1	Rn00572010_m1

**Table 2:** Changes in physiological parameters in response to pulmonary trunk banding (PTB) in male and female rats. Results are expressed as mean ± standard error of the mean (SEM). * *p* < 0.05 vs. Male-Sham group and ***^≠^*** *p* < 0.05 vs. Male-PTB group.

	Male-Sham (*n* = 6)	Male-PTB (*n* = 10)	Female-Sham (*n* = 6)	Female-PTB (*n* = 10)
Body Weight (Initial); g	109.2 ± 3.9	112.5 ± 2.2	106.3 ± 4.8	106 ± 2.8
Body Weight (Final); g	398 ± 16.4	381.4 ± 8.3	245.3 ± 5.5 *	238.2 ± 5.4 ^≠^
Liver; g	14.75 ± 0.89	13.22 ± 0.46	8.35 ± 0.35 *	8.29 ± 0.29 ^≠^
Lung; g	1.64 ± 0.10	1.66 ± 0.04	1.35 ± 0.06 *	1.21 ± 0.03 ^≠^
Spleen; g	1.03 ± 0.09	0.95 ± 0.08	0.73 ± 0.02 *	0.69 ± 0.02 ^≠^
Kidney; g	2.44 ± 0.10	2.32 ± 0.07	1.71 ± 0.06 *	1.60 ± 0.03 ^≠^
Tibia; mm	40.98 ± 0.29	40.50 ± 0.28	37.23 ± 0.19 *	37 ± 0.22 ^≠^

## Abbreviations

ANP	Natriuretic peptide A
BNP	Natriuretic peptide B
B2M	β-2-microglobulin
Col1	Collagen 1
Col3	Collagen 3
CO	Cardiac output
Ea	Arterial elastance
Eed	End-diastolic elastance
Ees	End-systolic elastance
ESP	End systolic pressure
F.I	Fulton index
FN	Fibronectin
HR	Heart Rate
LV	Left ventricle
PA	Pulmonary artery
PAH	Pulmonary arterial hypertension
PH	Pulmonary hypertension
PTB	Pulmonary trunk banding
RHF	Right heart failure
RV	Right ventricle
RVH	Right ventricular hypertrophy
RVSP	Right ventricular systolic pressure
SV	Stroke volume
TAPSE	Tricuspid annular plane systolic excursion
VTI	Velocity time integral
